# Precision public health—the Emperor’s new clothes

**DOI:** 10.1093/ije/dyy184

**Published:** 2018-09-12

**Authors:** David Taylor-Robinson, Frank Kee

**Affiliations:** 1 Institute of Psychology, Health and Society, The Farr Institute@HeRC, University of Liverpool, Liverpool, UK; 2 UKCRC Centre of Excellence for Public Health Research, Centre for Public Health, Queens University of Belfast, Belfast, UK

Recently the Centre for Disease Control suggested that ‘precision public health’ presents significant opportunities to improve the health of the population,^1^ but what does this concept add and does it live up to the hype? The promise is that by harnessing the power of Big Data, particularly genomic data, we may indeed see early gains in public health as a result of ‘more-accurate methods for measuring disease, pathogens, exposures, behaviors, and susceptibility’ to guide targeted prevention strategies.^2^ However, the term ‘precision public health’ is susceptible to misinterpretation. Long before Big Data in the form of personalized genetic and epigenetic profiling arrived, much public health screening and prevention strategy was premised on varying degrees of targeting and stratification, so this is nothing new. Nevertheless, others in the UK have used analogous terms such as ‘personalized prevention’ or ‘personalized public health’, representing them as part of an urgent agenda in which we must ‘reap the benefits of the genomic revolution’.^3^

The purpose of this article is therefore to highlight some of the evidentiary and philosophical challenges for the concept of ‘precision public health’ which have not been exposed to sufficient scrutiny. It is also to argue for a more considered focus beyond the genome, lest we career headlong towards a diversion of resources, away from what really matters, to the detriment of population health. To do this, we structure our critique by aligning it with the so-called population perspective on precision medicine (i.e. the ‘P4 approach*’*), namely that precision public health should aim to be Predictive, Preventive, Personalized and Participatory.^4^ The advantage of taking this approach is that whereas it focuses on the key dimensions and parameters of the decisions that could help improve care both for individuals and populations, it has gained traction among precision medicine proponents themselves. Thereafter we return to the more philosophical and ethical arguments that should remind us of a bigger picture and the trade-offs that we might be making by investing in ‘precision public health’.

## P1

The promise is that billions of data points will allow prediction of the future clinical needs of each patient, with a huge emphasis on how personal genomic and other ‘omics’ data, linked to population health datasets, might improve currently available prediction algorithms for chronic disease. The central idea here is that access to personalized genetic data might improve identification of higher-risk individuals and subsequently guide their lifestyle choices.^5^ Although there are undoubted public health benefits to harnessing population-level ‘big data’ to inform policy and evaluation, as epitomized by data linkage in the Nordic countries, risk stratification approaches are already well developed in clinical practice and public health on the basis of established risk factors. Evidence of the added predictive value of genetic markers is currently limited,^6^ but the inconvenient truth in the risk prediction industry is that we are predicting outcomes for groups of people like our patient**—**we are not predicting the outcome for an individual. On a very trite level of course, the outcome for any individual is binary, but there is a professional obligation to ensure that disease risks and chances of treatment benefit are communicated more effectively, made all the more challenging because of the enduring statistical illiteracy of many clinicians and patients.^7^

Predicting who will benefit from a particular intervention or change in behaviour at the individual level is challenging. An elegant demonstration of this notion appeared in a recent study by Finegold *et al*.,^8^ that used Monte Carlo simulation to evaluate how cardiovascular prevention benefits, in terms of life expectancy gains, might be distributed across a target group, dependent on their age, blood pressure, cholesterol or smoking habit. The authors showed that even for individuals starting with identical baseline risk, the outcomes are far from uniform, with a small unpredictable minority of people having dramatic gains, far more than the average for their risk stratum.^8^ Furthermore, for a risk factor or risk marker to serve as a useful discriminatory tool at the individual level, we need relative risks or odds ratios much greater than usually seen in epidemiology, greater than 50 or so.^9^ But in addition to this, the uncomfortable fact for companies who want to sell you your genetic profile, is that the predictive capacity of genetic variants is determined by the prevalence of the factors that interact with them, and so we need to question the portability of even the newer prediction engines driven by genetic data, at least as far as the personalization of prevention is concerned.^10^

## P2 / P3

Setting aside for a moment the challenges to developing better prediction engines, their use is premised on offering ‘personalized’ risk information to guide lifestyle choices and tailored prevention strategies (the second and third ‘P’s in the P4 approach). Unfortunately, we have little basis for optimism when we consider the evidence**—**providing genetic information to individuals has negligible impact on behaviour. In their updated 2016 systematic review, Hollands *et al.*^11^ comprehensively assessed the impact of providing DNA-based estimates of disease risk on health behaviours and motivation to change them, ultimately concluding that our expectations for such effects were not supported by the evidence. Hollands *et al.*^12^ are among a growing number of psychologists who doubt the utility of many current psychological theories of health behaviour change which privilege the reflective self and individual agency over more unconscious processes. Others go further and believe that behaviour change is characterized by highly non-linear phenomena, sensitive to initial conditions, and is best understood through the lens of complexity theory,^13^ but a problem for individual-level personalization is the fact that giving people ever more refined risk information seems to have little impact on behaviour and of course could cause harm.

We return to the place of individual agency and potential harms later, but one plausible conclusion that arises from this evidence might be that the risks have not been communicated properly. The controversy surrounding a paper from Tomasetti and colleagues^14^ in 2015, which was reported to infer that getting cancer was ‘a matter of luck’,^15^ testifies to the fact that communicating the distinction between aleatory uncertainty (the natural stochastic randomness in a process) and epistemic uncertainty (the scientific uncertainty in the model of a process due to limited data and knowledge), is subtle to many scientists, let alone the general public. The difference echoes Geoffrey Rose’s distinction between the causes of individual cases and the causes of incidence in populations. Cardiovascular risk prediction algorithms are used to inform patients of the chance that they will have a heart attack within the next 10 years, though at an individual level the truth is that no one knows whether a particular patient will experience an event or not. To address this, some psychologists have endeavoured to improve risk communication for health behaviour change by more accurately conveying the random component of risk at the individual level,^16^ using animation of random avatars in a Cates plot.^17^ Unfortunately, at least in the context of cardiovascular risk, this more progressive and honest way of conveying the risks resulted in even lower behaviour change intentions.^15^

## P4

The final P (Participatory) from the P4 personalized medicine mantra connotes the desire to fully involve the patient (or in our case, a healthy person) in making lifestyle choices. Now although a much greater research effort has so far gone into uncovering the physiological ways to personalized prevention (with genotypes and biomarkers), an equally valid approach to personalization is to better understand patient preferences. A great example of the importance of this was offered by Taksler *et al*.^18^ who appraised the effectiveness of a variety of preventive interventions and the time required for the benefits to be realized. They were able to rank the order of suitability for these interventions (in terms of gain in life expectancy) for a pair of very similar men (Bill and Adam), only one of whom had diabetes, but whose other risk factors were comparable. After a detailed analysis of the evidence, it turned out that lowering cholesterol and blood pressure might be more important for Bill than for Adam, whereas losing weight would gain more life expectancy for Adam. The point is that seldom have insights about individual preferences (which were not overtly solicited for Bill or Adam) been brought sufficiently into focus (for risk and benefits) for Personalized Prevention, and they may well overshadow the magnitude of the effects of any new molecular markers. Pravettoni and Gorini^19^ have argued that a P4 paradigm is inadequate if it does not also consider individual Psychology, advocating instead for a ‘P5’ approach that accounts for the way patients may make differing judgments of risk and benefits (which the Taksler study^18^ had not attempted). A similar perspective is offered by Rogowski *et al*.^20^ who have argued that treatment options could be tailored based upon on a more nuanced assessment of patients’ benefit-risk preferences and tolerability thresholds elicited through ‘Discrete Choice Experiments’. In this context, better assessment and communication of the potential harms of providing genetic information to patients and the general public are required, discouraging premature use of technologies with little evidence of health benefit, which potentially pose significant health and social risks.^21^^,^^22^

## The importance of social context

**Figure 1.**
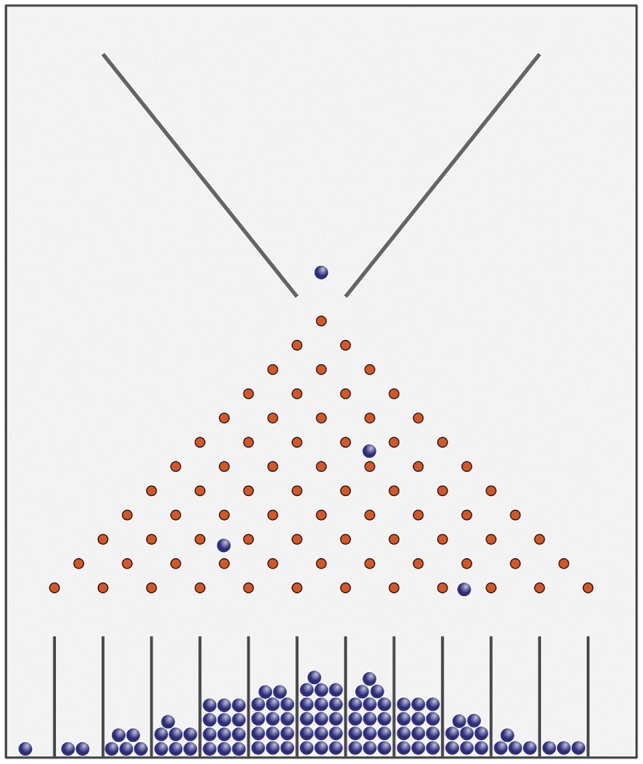
Galton's Quincunx. Sir Francis Galton invented the Quincunx, which demonstrates how a normal-distribution can be generated from a random process. Spherical beads or marbles are funnelled into the top where they collide with an array of pins, bouncing either left or right after each collision, finally collected into wells at the bottom. With the pins organised as per the figure, the height of the balls in each well generates a normal distribution. About 100 years later physicists were able to build two models of the Quincunx, one designed to generate the normal distribution and the other to generate the log normal distribution, by changing the arrangement of the pins. A simulation of the Quincunx is available here: http://www.mathsisfun.com/data/quincunx.html. This file is licensed under the Creative Commons Attribution-Share Alike 3.0 Unported license. Source: https://commons.wikimedia.org/wiki/File:Galton_Box.svg.

Now we have highlighted some specific empirical challenges to the P4/P5 approach to personalized prevention, but perhaps they are secondary to more fundamental philosophical and ethical problems. A key issue is the tendency for the personalization agenda to further entrench the individualization of risk, diverting public policy focus away from the upstream determinants of population health. Some leading scientists in our field have called for a move away from ‘risk factor’ epidemiology, to one that embraces a more nuanced complex systems perspective. Francis Galton’s Quincunx, which you see most days in the mechanism behind ‘The Tipping Point’ on daytime TV (see Box 1 and Figure 1), provides an example of how emergent phenomena arise as result of such complex systems. What structures the distribution generated by the Quincunx is not the innate qualities of the ‘elements’ themselves but the features of both the funnel and the pins**—**both their shape and their placement. Together, these structural features determine which pellets can (or cannot) pass through the pins and, for those that do, their possible pathways. Personalized public health risks placing too much emphasis on the elements cascading through the Quincunx (that is, for personalized public health, the people and their personal genetic and molecular profiles), rather than the system generating the distribution itself. As Krieger remarks:

Such an understanding of ‘structured chances’ is at odds with explanations of population difference premised solely on either determinism or chance, but forces us to consider the humanly engineered social systems … that give rise to the social stratification of disease risk.^23^

**Box 1. Galton’s Quincunx.**

Sir Francis Galton invented the Quincunx, which demonstrates how a normal distribution can be generated from a random process. Spherical beads or marbles are funnelled into the top where they collide with an array of pins, bouncing either left or right after each collision, finally collected into wells at the bottom. With the pins organized as per the figure, the height of the balls in each well generates a normal distribution. About 100 years later, physicists were able to build two models of the Quincunx, one designed to generate the normal distribution and the other to generate the log normal distribution, by changing the arrangement of the pins. A simulation of the Quincunx is available at[http://www.mathsisfun.com/data/quincunx.html].

Risk stratification and prediction models for poor child health and development outcomes have shown that maternal demographic, income and health behaviour data that can be easily collected at birth are the best predictors of longer-term outcomes.^24–27^ Furthermore, area deprivation has also been shown to significantly modify the performance of cardiovascular risk prediction models in adults.^28^ Using the elegantly simple example of the Broad Street pump, Khoury and Ioannidis suggest that a better use of routinely collected population data on upstream determinants of the distribution of disease is likely to be more fruitful than ‘omics’ data for improving risk prediction at individual and population levels.^29^ So when the Centers for Disease Control and Prevention (CDC) see the opportunity of genomics and precision public health to pave the way for enhancing our understanding of the genetic and epigenetic origins of inequalities,^1^^,^^30^ they seem to us to rather miss the point.

## A false dichotomy

Calls for a greater focus on upstream determinants in the Big Data enterprise are by no means new, but Diez Roux *et al*.^31^ highlight both the individual and the population benefits including: (i) enabling greater precision in diagnosis and improved treatment (through for example improved risk stratification and greater attention to patient context in treatment decisions); (ii) detecting patients with social or behavioural risk factors that, if addressed, might lower disease burden and improve management and recovery; and (iii) improving the capacity of health systems to tailor services to the needs of the population they serve. However, two further examples highlight how misguided it might be to try to create a false dichotomy between ‘personalized’ and ‘population-based’ approaches to prevention. Krypidemos and colleages, using a microsimulation approach, show that combining structural population-wide interventions with individual screening in the most deprived areas is most likely to maximize both effectiveness and equity of primary cardiovascular (CVD) prevention.^32^ Beheshti *et al*.,^33^ on the other hand, using an ‘Agent Based Modelling’ approach, show that targeting obesity interventions based only on individual characteristics is less effective at the population level than when the ‘invisible’ influence of social norms and social networks is taken into account.

Many commentators have expressed concerns that tailoring treatments to patients based on their individual genomes may lead to increasing inequality in human health.^34–38^ Some of the earlier discourse in the USA about the impact of personalized medicine and personalized prevention on socioeconomic inequalities has focused disproportionately on differential access to personalized medicine.^39^^,^^40^ More advantaged social groups may be more likely to benefit from the targeted development, uptake, affordability and access to personal genomic and pharmacogenetic testing technologies.^41–44^ For example, in the context of breast and lung cancer screening and treatment, poorer Black and ethnic minority groups are less likely to be recruited into trials or have potential genetic risk markers identified, and they are less likely to access new therapies when these are developed.^34^^,^^35^ It seems to us naively optimistic to imagine that interventions that rely on individual agency, behaviour change and compliance with treatment will have much impact on health inequalities, and they may even widen them.^45^

Thus if we are to reduce inequalities and maximize the benefits to individuals and populations, a whole-system and upstream approach to prevention should be combined with the so-called ‘personalized’ approach.^32^ However, the increasing burden of chronic ill health in adulthood, and the stark inequalities in healthy life expectancy in countries such as the UK, are partly because we have not successfully embedded and operationalized such approaches to addressing the social determinants of health across policy. There has long been a woeful mismatch in health and scientific funding favouring high-tech solutions to problems that could be prevented by allocating funding to addressing the wider social determinants of health over the life course. Despite the 5-year forward view for the NHS in England suggesting that a ‘radical upgrade in prevention and public health’ is required, only about 4% of the NHS budget is spent on prevention;^46^ and prevention research receives about 5% of all public spend on health research.^47^ Channelling limited resources into developing greater numbers of expensive treatments and diagnostic tools targeted at small segments of the population can only exacerbate this situation and divert attention from the bigger picture, namely what are likely to be more effective and ethical ways to improve population health and narrow inequalities. In this context, the contribution of personalized/stratified approaches for population health and health inequalities remains unclear, and risks diversion of both focus and resources away from the big picture.

## Funding

DT-R is funded by the MRC on a Clinician Scientist Fellowship (MR/P008577/1).

**Conflict of interest:** The authors have no involvements that might raise the question of bias in the work reported or in the conclusions, implications or opinions stated.
